# Developing a model for integrating sexual and reproductive health services with HIV prevention and care in KwaZulu-Natal, South Africa

**DOI:** 10.1186/s12978-018-0633-1

**Published:** 2018-11-15

**Authors:** Cecilia Milford, Fiona Scorgie, Letitia Rambally Greener, Zonke Mabude, Mags Beksinska, Abigail Harrison, Jennifer Smit

**Affiliations:** 1 MatCH Research Unit (MRU), Department of Obstetrics and Gynaecology, Faculty of Health Sciences, University of Witwatersrand, Durban, South Africa; 2 Wits RHI (Reproductive Health and HIV Institute), Faculty of Health Sciences, University of the Witwatersrand, Johannesburg, South Africa; 3 Department of Behavioral and Social Sciences, Brown University School of Public Health, Providence, RI USA

**Keywords:** Integration, Reproductive health, HIV, Family planning, South Africa

## Abstract

**Background:**

There are few rigorous studies evaluating the benefits of vertical versus integrated delivery of healthcare services, and limited published studies describing conceptual models of integration at service-delivery level in public healthcare facilities. This article seeks to fill this gap, by describing the development of a district-based model for integrating sexual and reproductive health (SRH) and HIV services in KwaZulu-Natal, South Africa.

**Methods:**

Baseline data were collected from seven urban public healthcare facilities through client and provider interviews, and a facility inventory was completed to assess current service integration practices. Feedback sessions were held with health providers from participating facilities to share data collected and explore appropriate integration scenarios. A conceptual model of potential service integration was then designed, and subsequently implemented and evaluated in the research sites.

**Results:**

Key principles of the model included a focus on health system strengthening and strong community input and involvement. The model was designed primarily to support the integration of family planning into HIV services, and included measures to improve client and commodity monitoring; capacity building through training and mentorship; and a ‘health navigation’ strategy to strengthen referrals within and between public healthcare facilities. Endline evaluation data were collected in the same facilities following implementation of the model.

**Conclusions:**

This manuscript demonstrates the utility of the conceptual model. It shows that service integration can be accomplished in a phased manner with support of community and healthcare providers. In addition, local context must be taken into account and the components of the model should be flexible to suit the needs of the health system.

## Plain English summary

There are few studies evaluating the benefits of vertical versus integrated delivery of healthcare services, and limited studies describing conceptual models of health service integration in public healthcare facilities. We describe the development of a district-based model for integrating sexual and reproductive health (SRH) and HIV services in KwaZulu-Natal, South Africa.

Before the development of the model, baseline data were collected from seven urban public healthcare facilities using client and provider interviews, and a facility inventory was completed to assess how existing services were being integrated, if at all. Feedback sessions were held with healthcare providers from participating facilities to explore possible scenarios for integrating services that could work in their settings. A conceptual model of service integration was then designed, implemented and evaluated in the research sites.

Key principles of the model included a focus on health system strengthening and strong community input and involvement. The model was designed primarily to support the integration of family planning into HIV services and included strategies to improve the monitoring of patients seen and commodities used (such as female condoms or contraceptive pills); capacity building of staff through training and mentorship; and a ‘health navigation’ strategy to strengthen referrals within and between healthcare facilities. Endline evaluation data were collected in the same facilities following implementation of the model.

The utility of the integration model we developed is explored in the manuscript. We show that service integration can be accomplished in a phased manner with the support of community members and healthcare providers. In addition, local context must be taken into account and any model used to guide integration of healthcare services should be flexible to suit the needs of the health system.

## Background

There has been a renewed focus on integrated health services in South Africa in the last 20 years [1]. This is largely in response to a growing unease about the verticalisation of HIV services, which resulted partially from the single-disease funding approach to HIV [2, 3]. Integration has also received attention thanks to an increasing desire to meet the sexual and reproductive health (SRH) needs of people living with HIV (PLHIV) [1, 4, 5]. More recently research demonstrating the teratogenic effect of Dolutegravir (first line antiretroviral regimen) on babies [6] has highlighted the need for integrated family planning and HIV services.

South Africa is a country where service integration in the fields of HIV and SRH is critically important. It has one of the largest populations of PLHIV globally, with a national HIV prevalence of almost 18% (in adults aged 15–49 years), in 2017 [7, 8]. The ‘rollout’ of anti-retroviral therapy (ART) was initiated by the South African government in 2005, and as of 2017, approximately 4.4 million South Africans were on ART [8]. While providing ART on this scale is challenging for the health system, and numbers on treatment still fall short of the UNAIDS 90–90-90 targets [8], remarkable success has been achieved in initiating clients on treatment. This initiation of treatment is affected by clients’ ability to reach health services and, once there, to negotiate a complex range of visits and medical tests. In some settings, multiple ‘stops’ within one facility are required, and some clients must move repeatedly between locations which may be situated some distance apart within the facility. In addition to HIV services, these clients need to utilise SRH services, which are often not matched to their particular needs and may not necessarily be offered within HIV clinics at primary healthcare facilities. Furthermore, clients accessing SRH services in health facilities are known to be at high risk of HIV acquisition, and therefore need to be offered integrated HIV services [1, 9].

National and international funding focus on HIV prevention and treatment was partly responsible for the verticalisation of services in South Africa, and resulted in a diversion of attention from (and neglect of) provision of comprehensive SRH services [10]. Consequently, family planning (FP) services have suffered, the range of available and accessible methods narrowed, and uptake and continuation of contraception remains a challenge. Despite recent introduction of the implant and retraining on the intrauterine device (IUD) in the public sector (post implementation of this study), unmet need for FP in South Africa continues to be high, at 18% (in married and sexually active unmarried women), in 2016 [11]. In addition, data from the 2012 South African National HIV Prevalence, Incidence and Behaviour Survey, demonstrated that two thirds (66.5%) of women (15–49 years) who had reported a pregnancy in the previous 5 years, had not intended becoming pregnant [12].

Despite the release and roll out of a national contraceptive policy and guidelines promoting contraceptive-HIV integration in 2014 [13–15], and a renewed emphasis on integrating services at facility level in South Africa, little progress has been made with SRH and HIV integration in practice. Adoption and implementation of these guidelines has been slow, and there has been limited development of policy on HIV-SRH service integration more broadly [14]. As a result, individual effort to integrate services at district or facility level may be ad hoc or relatively uncoordinated. Furthermore, there is a lack of indicators or agreed upon ways to measure integration [16–18]. In KwaZulu-Natal Province more specifically, the public healthcare services have been politically divided (District and Municipal systems), each with different administrations and budgets, which creates further challenges in service provision and integration.

Systematic reviews [17–20] demonstrate that integrating SRH and HIV services could yield a number of benefits, including increasing uptake of contraception, condom use, HIV testing, and services for prevention of mother-to-child transmission of HIV (PMTCT), along with more rapid ART initiation. However, some integration interventions have not been sufficiently evaluated [18, 20] and health, stigma and cost outcomes are not adequately reported on [19]. Furthermore, there remains little consensus on how to operationalise and best integrate these services [1].

In this manuscript we report on the development of a district-based model to provide comprehensive and integrated SRH and HIV services appropriate to the local context. At the time of this research, health services (including FP, HIV and other SRH services) in South Africa were being offered independently of one another, with few formal linkages between services. Although systems for referral are improving, even in healthcare settings that are under-resourced (often paper-based systems), under-staffed and highly burdened, tracking clients who are referred out of a facility is difficult, and missed opportunities for reaching clients with comprehensive SRH and HIV services continue to occur.

### Study aim and setting

Our aim was to improve uptake of services, but also to provide input to emerging South African policy on integrated services, by generating empirical evidence on the effectiveness and feasibility of an integration model in this setting. Working in an urban district, the eThekwini District in KwaZulu-Natal Province, South Africa, the study was located in seven healthcare facilities, including five primary healthcare clinics, a community health centre and a District level hospital. We aimed to reach young women in particular, who are at highest risk of HIV acquisition and have many unmet FP and SRH needs. KwaZulu-Natal had an HIV prevalence of 18.1% in 2017 [8], and women (15-49 years) in the province have continued high levels of unmet FP need (20.1% in 2016) [11]. We sought to explore innovative community-orientated strategies to reach this group and improve their access to SRH services.

In this manuscript we describe work done to inform the development of the integration model (study design), report briefly on baseline data used to inform the model (key findings from baseline research), describe the development and key components of the model (discussion: designing the model), and reflect on the main implementing challenges experienced and lessons learned for future SRH services integration (implementation successes, challenges and lessons learned).

Endline process evaluation data are discussed only where relevant to our reflections on the effectiveness of the model, but are reported on in more detail elsewhere [21]. Activities encapsulated within the integration model drew on simple, tried-and-tested methods as well as novel and innovative approaches to health systems strengthening, often adapted from contexts outside of HIV and SRH service provision.

## Methods

### Study design

#### Part 1

Key informant interviews (*n* = 21) were held in 2008 with individuals representing non-governmental organisations (NGOs), academia and the Department of Health (DoH) [22], to gather baseline data on understandings of ‘integration’, perceptions of current integration practices in South Africa, and pressing integration needs. Individuals with knowledge and experience of integrating SRH and HIV services in South Africa were purposively selected. Thereafter, snowball sampling was used to ensure information-rich cases were sampled and appropriate data were collected [22]. These data were qualitatively analysed using NVivo (QSR International).

#### Part 2

Key informant interviews were followed by baseline research comprising facility audits and interviews with healthcare providers and clients. More specifically, four focus group discussions (FGDs) were held with healthcare providers (*n* = 43), and a cross sectional survey was conducted with providers (*n* = 46), to explore current integration practices, and challenges and experiences with these. Healthcare providers were purposively selected to represent different categories of providers working in HIV and SRH services in all the participating facilities. In the FGDs, healthcare providers were grouped together according to senior management, nurses and doctors, and enrolled nurses and counsellors, to allow participants to speak freely [21]. Providers who participated in the baseline cross-sectional survey were from antenatal, FP, primary healthcare (PHC), HIV counselling and testing (HCT) and sexually transmitted infection (STI) services. Of all baseline survey participants, 89% were female, their mean age was 43.7 years, and their mean years of working were 18.3 years [21].

An exit interview in the form of a cross-sectional survey was conducted with clients (*n* = 269) accessing HIV or reproductive health related services (including HCT, antenatal, perinatal, PHC, STI and FP services), at the seven participating facilities in 2009. The client sample was selected by ensuring representation of clients attending the different SRH services in the facilities. Approximately 30–40 clients were enrolled per clinic, and approximately 50 clients at the hospital, and 82% of the clients were female. Client interviews explored services accessed and experiences with and attitudes towards integrated services. This information was supplemented by facility inventories conducted within the participating facilities, which enabled better understanding of the SRH, FP and HIV services offered at these facilities, and the extent to which these services were already integrated.

#### Part 3

Results from the baseline data (Parts 1 and 2) were explored, and used to inform the development of an integration model that responded to the needs and challenges specific to the context within which we were working. Specific integration gaps and challenges, as well as current practices that were identified by clients, providers and key stakeholders were focused on in order to develop a model that could be tailored to the specific healthcare facilities we were working in. This draft model was presented to different working groups which were established for the duration of the project. The purpose of the working groups was to inform the process of developing the integration model and to support its implementation. The groups included a District Working Forum (DWF), comprising operations and nursing service managers and nursing staff from the seven facilities. The DWF met twice a year throughout the project period and reviewed the proposed integration model, provided input to model development and implementation, assessed overall progress, and offered experiential advice and support. A Scientific Advisory Board (SAB), including academics, integration experts and managers in the local and municipal DoH, and a Community Advisory Board (CAB), which comprised of community health workers, NGO personnel, and other community role-players who represented the communities served by the health systems, also provided ongoing input to the model development and implementation process. In addition, the study team met regularly throughout the project with healthcare providers at the participating facilities to elicit input and provide iterative feedback on the integration model. Due to the frequent and interactive meetings with the groups, real time feedback was incorporated into development of the model.

#### Part 4

Following the implementation of the model, endline process evaluation data were collected (in 2011) via a cross sectional survey with providers (*n* = 44) and clients (*n* = 300), and facility inventories were conducted within participating facilities. Providers and clients who participated in the endline were sampled in the same way as for the baseline survey. Data were collected on the same parameters at baseline and endline to determine explore changes in data and determine usefulness and success of the model (and are reported elsewhere). Cross-sectional data were entered into SPSS versions 24 and 25, and descriptive statistics were calculated. Where necessary Pearson’s Chi-square and Fisher’s Exact Test were used to determine significance of change in variables over time.

This manuscript focuses on Part 3 – the development of an integration model, based on initial findings from the baseline research (as described in Parts 1 and 2).

### Study approvals and support

The study was approved by the University of the Witwatersrand’s Human Research Ethics Committee (M080624). The University of KwaZulu-Natal’s Biomedical Research Ethics Committee provided reciprocity approval. Provincial, District and Municipal DoH approval were obtained, along with site support from each of the participating healthcare facilities.

## Results

This manuscript describes the development of the integration model, based on initial findings from baseline research. Key baseline findings are presented here as they pertain to the model development, and more detailed evaluations, comparing baseline and endline results are reported elsewhere^1^ [21].

### Key findings from baseline research

Information elucidated from those involved both in providing and receiving services in the study facilities proved to be essential in orientating the model development process.^2^ Client survey data demonstrated that although clients were able to access the SRH services they required, they expressed some frustration with long waiting times – where almost a quarter (24.1%) at baseline did not agree that the waiting time was reasonable. Little integration of services appeared to be happening, and SRH services received by clients were not comprehensive. Few clients who had accessed the clinic for FP services on the day of the interview were offered other SRH or HIV services by a provider. Less than a third (29.7%) attending FP services had been offered HCT services on that day or at previous FP visits. However, communication about male condoms and dual protection was common, irrespective of the reason for the clinic visit. More than a third (34.1%) of clients reported having queued more than once in a single day for separate services at the same facility.

Clients indicated a preference for seeing the same provider on returning clinic visits, more than three quarters (77.3%) specified that if given a choice, they would like to see the same provider at each visit, suggesting that continuity of care is an important consideration when designing integrated services. This preference was also about the availability of a ‘one-stop shop’ for accessing PHC, HIV and FP services within the same facility, believed to result in shorter waiting times. Both healthcare providers and key informants expressed favourable attitudes towards the integration of HIV and FP services [21, 22], and some believed it would be more convenient for clients to access all the services they needed from the same provider [21]. Providers and key informants also wanted greater involvement of the community in service integration (including peer support systems), implying a potential extension of integration programmes beyond health facilities themselves.

Health providers expressed some concerns about the feasibility of integrating services, fearing an increase in workload and consultation times, making it difficult for them to see all clients coming to the facility in a single day [21]. An additional challenge anticipated by providers was the translation of new policies and guidelines into practice due to numerous operational barriers, such as shortage of resources – infrastructural, human and financial [21]. Perhaps because of these concerns, key informants stressed that integration of services should be flexible and tailored to specific contexts.

## Discussion: Designing the model

Our challenge was to design a model of integration that could meet the needs and challenges of this setting, but also be feasible, practical and affordable for the participating facilities. Based on key informant opinion [22] and a review of the integration literature, it was clear that our model needed to allow for internal variation between sites, and it also had to be relevant to the context of the broader health system. Our approach, therefore contained broad generic components, allowing for some adaptation at sites, recognising that “one size *doesn’t* fit all” [23].

In addition to a lack of agreed upon indicators to measure integration [16–18], another reason why it is difficult to evaluate the merits and drawbacks of integration efforts across different settings is that “integration” itself has been conceptualised in many different ways [10, 24]. Some have described it as a “spectrum” [25] or, as a “continuum rather than as two extremes of integrated/not integrated” [26]. Furthermore, integration has been characterised as “full” (multiple services offered on-site) versus “partial” (referral to off-site services) [27]. It can be facility level, provider initiated/driven, or client initiated/driven. There is a further need to identify the *direction* of integration: i.e. what new or existing services are being integrated into which existing programme or service? A WHO systematic review on linkages between SRH and HIV services, identified at least six possibilities: antenatal care clinics adding HIV services; HCT centres adding HIV services; FP clinics adding HIV services; HIV clinics adding SRH services; STI clinics adding HIV services; and PHC clinics adding HIV and/or SRH services [28].

In our study, decisions about direction of integration were informed by baseline findings that identified provision of FP and other SRH services to PLHIV as a neglected priority in this setting, which has more recently been highlighted by the teratogenic effects of Dolutegravir in early pregnancy [6]. We therefore designed a model that integrated FP into existing HIV services, and HIV into FP services, but which could ultimately be adapted to support a range of alternative configurations. Integrating FP into HIV services has numerous potential benefits. It creates opportunities for reaching men with FP information and services (for example in PHC related services), and for counselling serodiscordant couples and assessing the suitability of a contraceptive method for PLHIV, for instance in regard to potential drug interactions between contraceptive method and ART. It also allows for the promotion of dual protection and reiteration of FP messages, along with the resupply of FP methods to HIV clients during regular repeat visits [23]. On the other hand, integrating HIV services into FP services allows for HCT and HIV services to be provided to clients accessing FP methods.

In our model, these services were integrated at both provider and facility level. In the former, one provider would offer multiple services, while the latter involved mechanisms to support internal referral, either at the same visit or a future one. Measures to maintain effective linkages between services were also included to improve coverage and continuity of care.

Some challenges emerged during model development. These centred on questions of how to ensure genuine community involvement; of building capacity without over-relying on training; and of making a difference to the quality of services in an already over-burdened health system with high staff turnover. Additional challenges became evident when reviewing the literature on potential barriers to the effectiveness of integrated programmes. At the facility- and health systems-levels, such barriers could include: a lack of clear guidelines on which service is to be integrated in which department and how; inadequate integrated training for supervisors; and stock-outs of contraceptive supplies [4]. From the perspective of clients, integration could be less effective if they perceive integrated services to involve long waiting times and inadequate privacy; and to allow insufficient time for asking questions during consultations [29]. Overall, we wanted to anticipate potential barriers such as these and pre-empt them as far as possible.

**Fig. 1:**
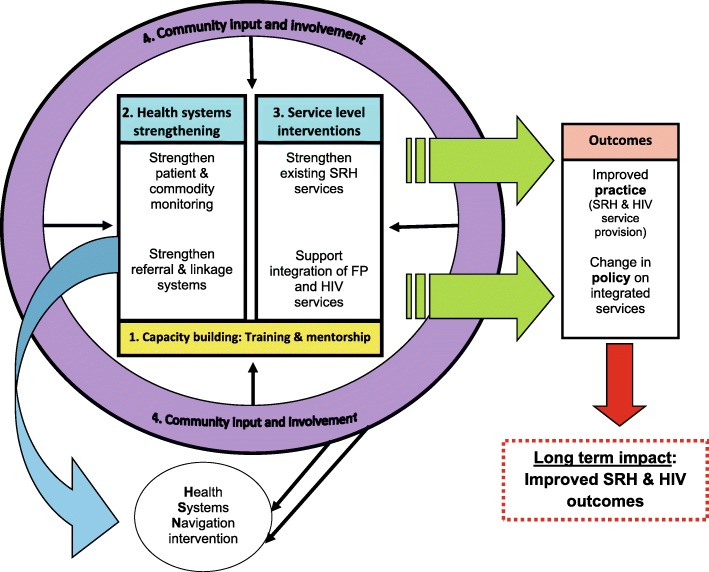
Conceptual representation of integration model developed for the eThekwini Integration Project

A preliminary integration model was initially presented to role-players from the SAB, DWF and CAB, and their input requested. Specifically, healthcare providers gave valuable input on what was realistic and reasonable within their facilities. The components and details of the model were adjusted based on these discussions. The final model comprised four inter-connected intervention areas, namely: capacity building; health systems strengthening; service level activities and interventions; and community input and involvement (see Fig. 1). Key expected outcomes included improvements in the practice of offering SRH and HIV services to clients – including greater efficiency, effectiveness and higher uptake of services – which in turn could facilitate recommendations for updating policies on integrated services. Each of these component intervention areas are described in more detail in the sections that follow.

### 1. Capacity building: Training and mentorship

Building the capacity of healthcare providers to offer integrated services was an essential component in the model. Capacity building also underpinned activities directed at health systems strengthening and interventions designed to improve the quality of individual services. Baseline research indicated that in many cases, only updates were needed on new policies or developments in the fields of HIV and SRH, rather than full training in basic material that was already familiar to providers. In each instance, HIV and SRH topics were addressed from the perspective of integrated services. Importantly, training was also provided on more ‘systemic’ issues, such as methods to strengthen referral systems, monitoring and evaluation (M&E) and record keeping. To increase attendance, training sessions were conducted on-site, in providers’ own facilities. They also included non-clinical staff (such as clinic receptionists and security guards), along with community representatives serving on the project’s CAB.

In the early stages of the project, many providers had indicated that training may have limited effect on practice in the long term, as providers return to work and seldom implement the theoretical knowledge learned during training. In anticipation of this, we conducted post-training mentorship in one facility. We used one-on-one mentorship to guide the application of new skills and knowledge, and to improve confidence to do this, once providers had returned to their posts. Support was also offered to the facility supervisor, equipping her with skills required to take ownership of the mentorship programme over time. Peer mentorship has been demonstrated as effective and feasible in capacity building for SRH and HIV service integration [30].

### 2. Health systems strengthening

Many of the challenges associated with implementation of integrated services stem from systemic weaknesses, where the health system itself is burdened by infrastructural, logistical, training and service management limitations [4]. While ‘fixing’ the health system was too ambitious, an integration model would be incomplete if it aimed only to combine services that had previously been offered vertically and did nothing to strengthen the functioning of the broader health system in which services were couched. Two key interventions were therefore included, to contribute to health systems strengthening: measures to improve client and commodity monitoring, and to improve referrals and linkages between services.

#### Improving information and supply chains: Client and commodity monitoring

Many challenges surround the recording of client data in public facilities in South Africa. Healthcare providers are required to enter client data by hand into multiple registers for each consultation, which is cumbersome and time consuming, and may inevitably lead to incomplete and inaccurate entries. Baseline data and planning discussions with stakeholders revealed the need for a single, streamlined register in which all client details could be captured [31]. Such a change however, was unfortunately beyond the scope of the project, as it would have required national DoH input and would have taken several years to implement.

Instead, we assessed the practice of record-keeping to identify shortcomings that could be targeted in future interventions. This involved a thorough review of registers and patient-held cards that were in use in the healthcare facilities at the time of the study. Providers were interviewed to build a picture of record-keeping overall, and to isolate the challenges from their perspective. On the basis of these data, we generated a list of recommendations to link monitoring systems more effectively with integrated practice and presented these to local and provincial health authorities in a ‘State of the Art’ dissemination meeting. Recommendations presented included reducing the number of registers while still capturing the same indicators that were already being recorded in a more integrated manner; formal training of healthcare providers on the practicality of the data and on understanding of the importance of the indicators being measured and monitored in clinic registers, to facilitate more reliable reporting; as well as the employment and training of dedicated data clerks to facilitate higher quality data capture.

Although we were unable to implement these recommendations as part of our model as this would have required extensive high level DoH input, which was not feasible within the study timeline, we were able to train providers to improve record-keeping practice within the existing system. In addition, regular feedback sessions were held in facilities to inform providers about client statistics in their facilities, which were generated at a provincial level, based on facility level data entry. These sessions highlighted incomplete data records and reinforced the importance and usefulness of accurate record keeping in facilities, and facilitated improvements in data record keeping.

Securing health commodities and ensuring that procurement is reliable has been identified as an often overlooked need in integration planning [28]. Based on an initial assessment of how FP commodities were being monitored in the study facilities, we designed a series of interventions to strengthen the supply chain overall and improve monitoring systems for procurement and re-supply to minimise stock-outs. Providers were trained to use these monitoring systems and were supported in their attempts to access stock from central distribution points. Support was provided to all facilities to ensure a continuous supply of contraceptives, particularly in a newly established HIV Wellness Centre linked to the antiretroviral (ARV) Clinic in one facility, which had no previous experience with stocking and ordering these commodities. In addition, a guidance note was developed to facilitate access to female condoms at facility level, and the numbers of female condoms distributed were measured throughout the project.

#### Innovative methods to improve referral & linkage systems

Our baseline data demonstrated that referrals at the study sites – both within and between facilities – were often unsystematic and uncoordinated, with no systems in place to monitor referral and follow-up success rates. To address these gaps, we adapted a ‘Health Systems Navigation’ intervention to improve referrals in this setting – a concept originating in cancer-treatment settings in the United States, but with the potential to be used in HIV service settings in southern Africa [32–34]. In this intervention, peer and other lay support workers were trained to assist clients in navigating complex HIV care and SRH services, thereby improving health outcomes (treatment initiation and/or adherence) and the quality of client experience and care.

We implemented the ‘health systems navigator’ intervention in one facility only (the District Hospital) in our setting. The aim was to increase client referrals within the SRH sector (FP, antenatal and postnatal care, abortion), and between designated HIV services (ART, PMTCT, HCT), and to improve clients’ overall experiences when accessing healthcare. Aside from making integrated services part of routine practice, this innovation aimed to help break down community members’ fears of asking for help in the health system and their reluctance to request specific SRH services owing to stigma.^3^

Navigators were trained in several roles. They gave health talks to clients in facility waiting areas and at community level events on a wide range of SRH, FP and HIV topics, while also offering information on the availability of integrated services. Navigators escorted clients to service points where needed, and actively followed up referrals afterwards to ensure that clients had successfully accessed relevant SRH and HIV services. Both clients and healthcare providers provided favourable feedback on the inclusion of health navigators in the integration model. The navigator role was structured to be comparable with the local Community Health Worker skills level and remuneration, in order to ensure operational feasibility and facilitate future sustainability should the model be scaled up.

### 3. Service-level interventions

With these health systems strengthening interventions in place, our focus turned to address the specific services targeted for integration. Our intention was to introduce FP services into ARV clinics, HIV Wellness Centres, Well Baby and PMTCT clinics – all of which had previously provided limited FP services to clients or none at all. This integration at service-level was kick-started by a series of training, or capacity-building, activities and consolidated through an expansion of existing SRH services.

#### Supporting integration of FP and HIV services

Effective integration of FP and HIV services required an element of task shifting. HIV service providers (counsellors, ARV providers, and PMTCT providers) were trained to also counsel their clients on FP needs and other SRH concerns. Furthermore, we trained FP service providers to perform HCT in sites where there were too few dedicated HIV counsellors.

Training was structured to respond to the particular needs of individual facilities, rather than offered as a generic package. In spite of this tailored, specific approach, however, many of the sessions across facilities turned out to be standard, and covered a variety of SRH and HIV topics, including: integrated female condom training; HCT and FP integration; ARV services and FP integration; expanding the FP method choice; and counseling clients on dual protection. All levels of healthcare providers were invited to participate in the training in order to facilitate integration practices both within and between healthcare facilities. Several job aids were used in this training, including female pelvic models and male condom models, the national guidelines on contraception, and information, education and communication (IEC) materials on emergency contraception, female condom use, and medical male circumcision. To ensure that an expanded method choice was being offered to clients, we also trained providers in the use of the WHO Reproductive Choices Flip-chart [35] and supplied them with copies of this tool for use during consultations. The flip-chart is designed to strengthen providers’ ability to offer clients a wider selection of contraceptive methods, encourage them to choose the most acceptable contraceptive method, and address broader SRH issues.

#### Expanding and promoting existing SRH services

Creating and implementing the integration model offered an opportunity to push the boundaries of existing SRH services, reconfiguring old services or even introducing wholly new ones. In addition to expanding the repertoire of FP methods offered to clients, in one facility we worked with healthcare providers to facilitate promotion of emergency contraception and to make it more accessible – by extending the service over weekends, when demand was likely to be higher. By implementing this in one facility only, we were able to explore the feasibility of expanding SRH services in other sites where there is operational feasibility and provider support.

IEC messaging was an integral part of the strategy to build awareness of the full range of SRH services on offer in facilities, particularly where they had not previously been offered. We used messaging printed on t-shirts, posters and pamphlets to promote dual protection, emergency contraception, female condoms and medical male circumcision, all couched within a broader context of integrated services. Health systems navigators wore these t-shirts, which not only made them visible to clients but also helped to communicate the availability of multiple services at the facility.

### 4. Getting everyone on board: Engaging the community

Community input and involvement was integral to the process of intervention design. Through the CAB and DWF, sustained input was elicited from community members and key stakeholders. While the CAB drew its members largely from clinic volunteers, NGO personnel and other community role-players, the DWF comprised mostly senior facility staff. Their participation in the study extended beyond feedback sessions, however, to include involvement in a series of broader local health-promotion activities. Through these forums, we sought buy-in from facilities and DoH, and built networks and relationships with community members and health authorities on the ground, harmonising our collective efforts to improve SRH and HIV services in the district.

Training in integrated services, expanded SRH services and promotion of female condoms was offered to members of the CAB, who readily accepted this opportunity to become better equipped to play active roles in their communities and facilities. Once NGOs, identified by CAB members as being active in areas around the study sites, had been mapped, we were also able to invite members of relevant NGOs to participate in training sessions. Finally, health systems navigators linked up with local NGO representatives during community outreach activities, joining them at community events to give talks on various SRH topics and raise awareness of the newly integrated services available.

### Implementation successes, challenges and lessons learned

A ‘success story’ from our study was the introduction of health systems navigators, which represented a possible solution to the difficulties around task-shifting and around changing scopes of practice. This component of the model demonstrated that there is strong potential for a new cadre of staff to take on multiple tasks and facilitate integrated services within South Africa’s over-loaded public healthcare system. The health systems navigators active in our study facilities managed to relieve some of the nurses’ workload: nurses reported that time had been freed up for them to focus on both clinical and administrative duties, making this a promising intervention that could be further developed and scaled up. The health systems navigators were utilised by nurses for activities beyond their original scope of work, including filing and other administrative tasks. Furthermore, since the conclusion of our integration study, navigators have been funded in the Province of KwaZulu-Natal by external funders (such as Pepfar), and are now working in SRH services in public healthcare facilities [34, 36].

In addition, the creation of a DWF was a particularly successful project component. Including healthcare providers from all levels of participating facilities (operational, managerial and nursing staff), enabled project buy-in and support. The regular meetings facilitated real-time contributions to the model development and implementation, and ensured that challenges could be addressed timeously if necessary.

Several obstacles made it difficult to fully implement all components of the model or to do so as originally planned. We found that – unsurprisingly – integrated service delivery takes time to establish and support. Buy-in and approvals from some partners took longer than anticipated, and required enormous investment into building relationships. We learned early on that it was essential to stagger the process of introducing new practices and not bombard facilities with activities all at once. As with any process of initiating institutional change, internal resistance must be acknowledged and accommodated, with appropriate sensitivity to the existing workloads of providers, which are often substantial.

It was sometimes difficult to get full attendance of providers at training sessions due to numerous factors such as heavy workloads, high turnover of staff and multiple commitments (such as staff meetings and district training programmes). Training was conducted on-site at hours convenient to healthcare providers and frequently rescheduled in order to address this challenge – but it remained difficult throughout implementation, and possibly diminished the impact of the intervention overall.

Despite our efforts to improve commodity monitoring and procurement, there were also frequent stock-outs, specifically of female condoms. This may have been because facilities were too busy to prioritize female condom planning, amidst competing demands on their time and other resources. Unfortunately, we also discovered that our involvement in facilitating procurement of female condoms ironically became a disincentive for facility staff to take on this task themselves.

An important lesson learned during capacity building activities was that it was important to know how flexible providers’ ‘scope of practice’ was prior to training them. Without this, the promotion of task-shifting was virtually impossible. One example was the attempt to train enrolled nurses and nursing assistants to counsel clients on the importance of contraception and to perform HCT, but HIV and contraceptive method counseling was not considered to be part of their formal ‘scope of practice’, therefore they were often not confident enough to counsel clients.^4^ In another example, we wanted to involve support staff (security guards, clerks, and nursing assistants) in more innovative ways and task-shift some integration responsibilities to them. Clerks in particular were identified as being under-utilised in facilities. Since we were not permitted to propose changes to job profiles in the research sites, these intentions could not be realised, although the initiative is nonetheless recommended for future integration efforts. An important outcome of this study is the finding that clerks and other healthcare facility support staff could do more, but that standard job descriptions and expectations may prevent this from happening. However, in our study, security guards were trained on services provided and were able to direct clients to these services and to condom dispensers located outside facilities, after hours.

A few interventions were clearly needed in this setting but fell outside of the project’s scope or were simply not pragmatic to implement. Firstly, more substantive changes were needed in the area of client flow and consultation hours. In all facilities, clients were only seen in the mornings (on a ‘first-come-first-served’ basis), as healthcare providers generally used the afternoons to catch up on administrative duties. If clients had to be referred for any additional critical care, this could also only happen in the mornings. Clients consequently arrived very early in the morning in order to secure their place in the queue, and often ended up waiting several hours to be seen. The provision of additional, integrated services to clients could potentially increase the duration of each consultation, leading to even longer waiting times for clients. In our context, extending consultation times beyond the mornings would certainly have lessened waiting times – but it was not possible for several reasons. Most clients depended on public transport, which is more limited later in the day compared to early mornings in this setting. Furthermore, providers themselves were reluctant to see clients in the afternoons, even if this meant that client-flow continued to be slow with rushed consultation times. Instituting human resource-level interventions to reduce waiting times – such as enabling flexi-hours and the use of shifts – were therefore omitted from the model, even if they made sense in theory.

Secondly, we wanted to implement a patient card system to facilitate improved record-keeping and linkages between services. This method of managing client data could be used as a tool to empower women to take control of their own health. For example, patient cards could capture multiple information including HCT, HIV status and CD4 count, in addition to serving as an FP tool. However, it requires a comprehensive tracking system, and needs to be consistently used to be effective. Introducing such an intervention would have needed buy-in at a higher level, as it would have had implications for wider DoH activities across the province and beyond, but it remains an important intervention for future consideration.

Finally, we identified and drafted a plan for an adolescent SRH clinic to be offered at one site, offering specific clinic times for school-going youth, dedicated FP queues for adolescents, and similar interventions. Due to unforeseen delays with ethics application processes, this was never implemented. It was viewed as an acceptable intervention and would be critical in future integration scenarios, in order to be able to provide multiple services to this vulnerable population.

Despite the fact that this model was implemented some time ago, and that supportive integration policies have been implemented since this study was undertaken, integration in practice has been slow [1, 5, 14]. Therefore, findings from this study remain useful to inform future integration policies and practices in this this setting.

## Conclusions

The project demonstrated that even a multi-faceted, complex model of integration can be implemented in low-resource, high-burden public healthcare systems, if it is done in a phased manner with buy-in and support of both community and healthcare providers. The process of design and implementation needs to take local context and facility level into account, and should be flexible to suit the needs of both the health system and the clients in order to have optimum effect. There need to be linkages between multiple health systems functions and components, including policy, financing mechanisms, supply chain management, and healthcare worker training [5]. We were able to present a draft integration strategy to stakeholders and policy makers at national, provincial and district DoH levels, that drew on empirical data from implementation of the model. Out of this experience, we made various recommendations for policy and planning. These recommendations include three key priority areas. Firstly, to develop integrated training and mentoring programmes – prioritise, revive and strengthen existing programmes, promote community training and use innovative methods (such as health systems navigation) to support this initiative. Secondly, to ensure a basic minimum package of integrated services, tailored to individual facilities, with standardised service points for integration, and integration indicators. Finally, to ensure communities are involved and engaged in integration activities, with a focus on hard to reach groups such as males and adolescents -using a bottom up approach. It is important to note that change is slow, and any suggestions for change require negotiation over time. The South African government is currently prioritising “putting integration into practice” [1], and our findings and recommendations have the potential to inform future policy and practice in this field.

## Abbreviations

ART: Antiretroviral therapy
ARV: Antiretroviral
CAB: Community advisory board
DoH: Department of Health
DWF: District working forum
FP: Family planning
HCT: HIV counselling and testing
IUD: Intrauterine device
NGO: Non-governmental organisation
PLHIV: People living with HIV
PMTCT: Prevention of mother-to-child transmission of HIV
SAB: Scientific advisory board
SRH: Sexual and reproductive health

## Footnotes

1 Additional analyses have been submitted for publication elsewhere.

2 The detailed results of the baseline and endline research will be published elsewhere, and are not presented in this manuscript, which focuses solely on the integration model. Key baseline findings are highlighted here to demonstrate findings which guided the model development.

3 The health systems navigation component will be published elsewhere.

4 Enrolled nurses or staff nurses are limited in scope of work by the Nursing Act (Act 33 of 2005), to only provide nursing care and treatment under the supervision of a professional nurse.
